# Up-regulated microRNA-143 in cancer stem cells differentiation promotes prostate cancer cells metastasis by modulating FNDC3B expression

**DOI:** 10.1186/1471-2407-13-61

**Published:** 2013-02-05

**Authors:** Xinlan Fan, Xu Chen, Weixi Deng, Guangzheng Zhong, Qingqing Cai, Tianxin Lin

**Affiliations:** 1 Center of medical research, Sun Yat-Sen memorial hospital, Sun Yat-Sen University, Guangzhou, 510120, China; 2 Department of Urology, Sun Yat-Sen Memorial Hospital, Sun Yat-Sen University, Guangzhou, 510120, China; 3 Department of Medical Oncology, Cancer Center, Sun Yat-sen University, Guangzhou, 510060, China; 4 Key Laboratory of malignant tumor gene regulation and target therapy of Guangdong Higher Education Institutes, Sun Yat-sen University, Guangzhou, 510120, China

**Keywords:** miR-143, Prostate cancer, Cancer stem cells, Differentiation, Metastasis, FNDC3B

## Abstract

**Background:**

Metastatic prostate cancer is a leading cause of cancer-related death in men. Cancer stem cells (CSCs) are involved in tumor progression and metastasis, including in prostate cancer. There is an obvious and urgent need for effective cancer stem cells specific therapies in metastatic prostate cancer. MicroRNAs (miRNAs) are an important class of pervasive genes that are involved in a variety of biological functions, especially in cancer. The goal of this study was to identify miRNAs involved in prostate cancer metastasis and cancer stem cells.

**Methods:**

A microarray and qRT-PCR were performed to investigate the miRNA expression profiles in PC-3 sphere cells and adherent cells. A transwell assay was used to evaluate the migration of PC-3 sphere cells and adherent cells. MiR-143 was silenced with antisense oligonucleotides in PC-3, PC-3-M and LNCaP cells. The role of miR-143 in prostate cancer metastasis was measured by wound-healing and transwell assays in vitro and bioluminescence imaging in vivo. Bioinformatics and luciferase report assays were used to identify the target of miR-143.

**Results:**

The expression of miR-143 and the migration capability were reduced in PC-3 sphere cells and progressively increased during sphere re-adherent culture. Moreover, the down-regulation of miR-143 suppressed prostate cancer cells migration and invasion in vitro and systemically inhibited metastasis in vivo. Fibronectin type III domain containing 3B (FNDC3B), which regulates cell motility, was identified as a target of miR-143. The inhibition of miR-143 increased the expression of FNDC3B protein but not FNDC3B mRNA in vitro and vivo.

**Conclusions:**

These data demonstrate for the first time that miR-143 was up-regulated during the differentiation of prostate cancer stem cells and promoted prostate cancer metastasis by repressing FNDC3B expression. This sheds a new insight into the post-transcriptional regulation of cancer stem cells differentiation by miRNAs, a potential approach for the treatment of prostate cancer.

## Background

Prostate cancer is the most frequently diagnosed malignant disease in men and the second leading cause of cancer deaths in US [1]. The treatment of prostate cancer with surgical resection, which may be combined with chemotherapy, hormone therapy or radiation therapy, is curative in many patients. However, most patients eventually relapse with castration-resistant prostate cancer and develop metastatic disease, which has a poor prognosis because no effective treatments are currently available [2]. Although basic knowledge related to metastasis has increased recently, many of the key elements remain largely unknown.

Cancer stem cells (CSCs), a small subpopulation of cells in a tumor, can self-renew and differentiate into multiple lineages, and they possess strong tumor-initiating capacity. Therefore, CSCs are thought to be responsible for tumor initiation, progression, therapy resistance, relapse and metastasis [3]. Accumulating evidence has demonstrated the existence of prostate cancer stem cells, which can be been enriched by sorting for CD44+/CD133+ expression [4,5] or by selecting cells that have the capacity to exclude Hoechst dye [6] or form spheres in serum-free suspension culture [7,8]. However, the role of prostate cancer stem cells in tumor development and metastasis is still poorly understood.

MicroRNAs (miRNAs) are small, noncoding RNAs of approximately 19 to 25 nucleotides in length that usually bind to the 3′-untranslated region (UTR) of their mRNA targets, resulting in degradation or translation repression [9]. Emerging evidence shows that the dysregulation of miRNAs is involved in cancer proliferation, differentiation, apoptosis and metastasis, and miRNAs function as oncogenes or tumor suppressors [10]. In addition, miRNAs have emerged as important regulators of CSCs. Yu and colleagues discovered that let-7 regulates breast tumor-initiating cells properties by silencing genes involved in self-renewal and differentiation [11]. A recent study has revealed that miR-34a inhibits prostate cancer stem cells and metastasis by directly repressing CD44 [12]. It was demonstrated that miR-320 suppresses the stem cell-like characteristics of prostate cancer cells by down-regulating the Wnt/beta-catenin signaling pathway [13].

A series of miRNAs has been identified to be up-regulated in prostate cancer, including miR-21, miR-24, miR-32, miR-125b, and miR-221/222. Conversely, miR-7, miR-34a, miR-101, miR-143/145, and let-7a have been identified to be down-regulated in prostate cancer [14]. Furthermore, several miRNAs have been identified as mediators of metastasis in prostate cancer. It was demonstrated that miR-221 was progressively reduced in aggressive and metastasis prostate cancer and predicted clinical recurrence [15]. A previous study revealed that miR-21 was overexpressed in prostate cancer and promoted apoptotic resistance, invasion and metastasis by targeting MARCKS [16]. A recent study has shown that miR-143 plays an important role in prostate cancer proliferation, migration and chemosensitivity by suppressing KRAS [17]. More recently, miR-29b was identified as an anti-metastatic miRNA for prostate cancer cells by regulating epithelial–mesenchymal transition (EMT) signaling [18]. However, the role of miRNAs in prostate cancer stem cells and metastasis remains to be elucidated.

In our previous study, we enriched and characterized prostate cancer stem cells from PC-3 sphere cells in a defined serum-free medium [7]. In this paper, we use spheres as a prostate cancer stem cells model to elucidate the role of miRNAs in prostate cancer metastasis. In this study, we compared the miRNA expression profiles of PC-3 spheres and adherent cells of prostate cancer and identified that miR-143 was related to CSCs and metastasis. Moreover, we demonstrated that the down-regulation of miR-143 suppressed migration and invasion in vitro and tumor metastasis in vivo.

## Methods

### Cell culture

The cell lines used in this study included the human prostate cancer cells PC-3, PC-3-M and LNCaP and SV40-transformed kidney cell line 293 T (ATCC, Manassas, VA). The prostate cancer cells were cultured in RPMI-1640 medium and 293 T were cultured in DMEM (Gibco, Invitrogen) supplemented with 10% FBS (Hyclone). Cells were grown to 90% confluence, trypsinized, and plated at a density of 1,000 cells/ml in serum-free DMEM/F12 medium (Gibco, Invitrogen) containing 20 ng/ml epidermal growth factor (EGF, R and D Systems, MN), 5 μg/ml insulin, 0.4% bovine serum albumin (Sigma, St. Louis, MO), and 2% B27 (Invitrogen, CA) in 10 cm^2^ culture dishes. To propagate spheres in vitro, spheres were collected by gentle centrifugation, dissociated to single cells, and cultured to generate the next generation of spheres [7]. Cells were grown in a humidified atmosphere of 5% CO_2_ at 37°C.

### miRNA microarray

Total RNA samples were analyzed by Chipscreen (Chipscreen Biosciences, Ltd., Shenzhen, China) for miRNA microarray experiments. Procedures were performed as described in detail (http://www.chipscreen.com). Each miRNA microarray chip contained 1,199 probes, including 703 identified human miRNAs and 393 predicted miRNAs. The arrays were scanned with a GenePix Pro 6.0 (Axon, Ltd), and images were analyzed using DMVS (Chipscreen Biosciences, Ltd.). Microarray data for each sample were normalized to the median. All data is MIAME compliant and that the raw data has been deposited in a MIAME compliant database (GEO, accession ID: GSE44069).

#### Quantitative RT-PCR

Total RNA was extracted from cells or tumor tissue of nude mice using Trizol reagent (Invitrogen) according to the manufacturer’s protocol. Total RNA was used for reverse transcription with the PrimerScript RT-PCR kit (TaKaRa Biotechnology, Dalian, China). MiRNAs were reverse transcribed using sequence-specific stem-loop primers (Invitrogen). Quantitative RT-PCR was conducted using a standard SYBR Green PCR kit (Roche) protocol with a LightCycler 480 real-time instrument (Roche). The relative expression was calculated using the 2^-dCt^ method. The transcription levels of GAPDH or U6 were used as an internal control. All specific Primers are listed in Table 1.

**Table 1:** Oligonucleotide Sequences for (q)RT-PCR and miR-143

	**Name**	**Sequence**
Oct4	Sense primer	5^′^-TCCCATGCATTCAAACTGAGGTGC-3^′^
	Antisense primer	5^′^-AACTTCACCTTCCCTCCAACCAGT-3^′^
Sox2	Sense primer	5^′^-TGGACAGTTACGCGCACAT-3^′^
	Antisense primer	5^′^-CGAGTAGGACATGCTGTAGGT-3^′^
Nanog	Sense primer	5^′^-AAGGTCCCGGTCAAGAAACAG-3^′^
	Antisense primer	5^′^-CTTCTGCGTCACACCATTGC-3^′^
GAPDH	Sense primer	5^′^-GCACCGTCAAGGCTGAGAAC-3^′^
	Antisense primer	5^′^-TGGTGAAGACGCCAGTGGA-3^′^
miR-143	Stem-loop	5^′^-GTCGTATCCAGTGCGTGTCGTGGAGTCGGCAATTGCACTGGATACGA-Ctgagcta-3^′^
U6	Sense primer	5^′^-ACACTCCAGCTGGGTGAGATGAAGCACTGTAG-3^′^
	Antisense primer	5^′^-CTCAACTGGTGTCGTGGA-3^′^
FNDC3B	Sense primer	5^′^-GCTTCGGCAGCACATATACTAAAAT-3^′^
	Antisense primer	5^′^-CGCTTCACGAATTTGCGTGTCAT-3^′^
miR-143	Sense primer	5^′^-GGGACAGACACCCGTTTTGA-3^′^
	Antisense primer	5^′^-GTGTTGCCCACGGTAATGCT-3^′^
	inhibitor	5^′^-GAGCUACAGUGCUUCAUCUCA-3^′^
	negative control	5^′^-UUCUCCGAACGUGUCACGUTT-3^′^

### Transient transfection

The transfection of PC-3, PC-3-M, and LNCaP cells with 50 nM miR-143 inhibitor or negative control (NC) (GenePharma, Shanghai, China) was performed with lipofectin 2000 (Invitrogen) according to the manufacturer’s protocol for 48 hours. The sequence of the miR-143 inhibitor and NC are listed in Table 1

### Stable miRNA expression cell lines

Lentiviruses containing GFP-miR-143 inhibitor or GFP-negative control miRNA vector were purchased from Genepharma. PC-3-M cells were pre-seeded in a 6-well plate overnight and infected with 200 μl of virus. 24 hours after addition of viruses, infected cells were selected by adding 20 ng/ml puromycin to growth medium for 4–5 passages. Stable cell lines were verified by qRT-PCR and fluorescence microscope.

### Transwell assay

The transwell assay was done by using transwell chamber consisting of 8 mm membrane filter inserts (Corning). The spheres were collected by gentle centrifugation and trypsinized to single cells in Trypsin-EDTA solution as well as the adherent cells. The cells were suspended in serum-free RPMI-1640 medium and counted by automated cell counter (Countstar). Then 5 × 10^4^ cells were added to the upper chamber, whereas lower chamber were filled with complete medium. After 12 hours of incubation, the cells in the upper chamber were carefully removed with a cotton swab, and the cells that had migrated through the membrane to the lower surface were fixed with 90% methanol and stained with 0.1% crystal violet. The invasion assay was performed according to a similar method, except that Matrigel (BD Biosciences) was pre-coated on the membrane of the upper chamber. The cell count was done under the microscope (200×).

### Wound-healing assay

One day before scratching, the cells transfected with miR-143 inhibitor or NC were seeded into 6-well plates to almost total confluence in 24 hours. An artificial homogenous wound was created onto the monolayer with a sterile 10 μl tip. After scratching, the cells were washed with serum-free medium. Images of cells migrating into the wound were captured at 0 and 24 hours by inverted microscope (100×).

### In vivo models of prostate cancer metastasis

All of the animal care and experimental procedures were approved by the Institutional Animal Care and Use Committee of Sun Yat-sen University. Male BALB/c athymic nude mice (4–6 weeks old) were purchased from the Experimental Animal Center of Guangdong province and housed in SPF barrier facilities under a 12 h light/dark cycle. PC-3-M cells that stably expressed miR-143 inhibitor (5 × 10^6^) or NC (5 × 10^6^) were suspended in 100 μl PBS and subcutaneously injected into the right side of the posterior flank of the mice. Ten mice were used in each group. Tumor development and metastases were monitored with a bioluminescence imaging system (ZKKS-MulAurora PI1024) on day 30 post-injection. Mice were photographed under 470/30 nm illumination, and images of the emitted fluorescence were acquired at 545/60 nm. Luminescence data were gathered over the maximum exposure period without pixel saturation (0.5–12 seconds).

### Bioinformatics

Potential miRNA targets were predicted and analyzed using two publicly available algorithms, including PicTar (http://pictar.mdc-berlin.de) and TargetScan (http://www.targetscan.org/). These searchable websites predict biological targets of miRNAs by searching for the presence of conserved 8mer and 7mer sites that match the seed region of miRNA and provide details 3′ UTR alignments with predicted sites. To decrease the number of false-positive results only putative target genes predicted by at these two programs was accepted.

### Luciferase reporter assays

The 3^′^-UTR luci vector was constructed using the psi-CHECK2 luciferase reporter vector containing a fragment of the FNDC3B mRNA 3^′^UTR, which carries a putative miR-143 complementary site. 293 T cells (5 × 10^4^ per well) were pre-seeded in a 24-well plate the day before transfection and transfected with 0.5 μg of the 3^′^ UTR luci vector and 50 nM miR-143 mimics or negative control using Lipofectamine 2000 (Invitrogen). Assays were performed using the Dual-Luciferase Reporter Assay System (Promega) after 48 hours of transfection.

### Western blotting

The total protein extracted from the samples was resolved on 10% sodium dodecyl sulfate–polyacrylamide gels and electrophoretically transferred to a polyvinylidene fluoride membrane. Blots were blocked with 5% skim milk followed by incubation with antibodies specific for either FNDC3B (Sigma) or GAPDH (Cell Signaling). The blots were then incubated with goat anti-rabbit or anti-mouse secondary antibody (Cell Signaling) and visualized using enhanced chemiluminescence.

### Statistical analyses

Data were presented as the means ± SD from three separate experiments. The differences between groups were analyzed using Student’s *t* test when only two groups were compared or a one-way analysis of variance (ANOVA) when more than two groups were compared. The differences between groups of metastasis in vivo were analyzed using Chi-squared test (*χ*^2^ test). All of the statistical analyses were performed with SPSS 16.0. The difference was considered to be statistically significant at P <0.05.

## Results

### MiR-143 expression was progressively increased during PC-3 sphere cells differentiation

**Figure 1:**
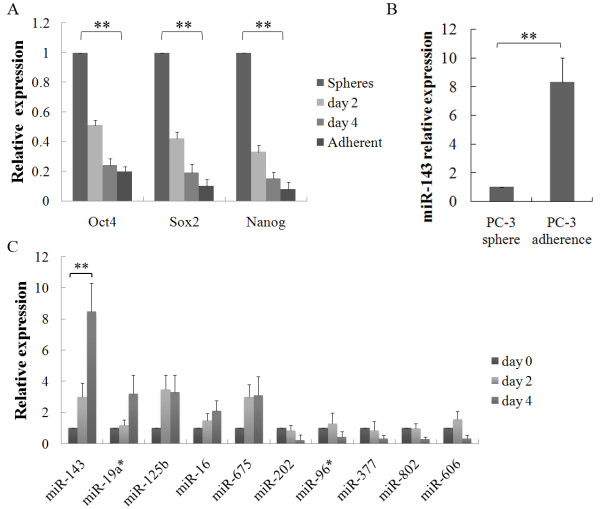
**MiR-143 expression was progressively increased during PC-3 spheres differentiation.A**, The relative expression of Oct4, Sox2 and Nanog were analyzed in PC-3 spheres, adherent cells, and re-adherent cells on days 2, and 4 by qRT-PCR (ANOVA). **B**, The relative expression of miR-143 in PC-3 spheres and adherent cells was determined by qRT-PCR (Student’s *t* test). **C**, The relative expression of miRNAs were analyzed on days 0, 2, and 4 after PC-3 sphere cells were digested into single cells for re-adherent culture by qRT-PCR (ANOVA) **p < 0.01.

First, to elucidate whether the PC-3 sphere cells turned into differentiated cells when sphere cells were digested into single cells for re-adherent culture (10% FBS-RPMI-1640 medium), we compared the expression levels of cancer stem cells markers, such as Oct4, Sox2 and Nanog by qRT-PCR. The expression of Oct4, Sox2 and Nanog were gradually decreased in re-adherent culture (Figure 1A). This suggested that PC-3 sphere cells had the cancer stem cells phenotype and differentiated in re-adherent culture. Second, to investigate whether miRNAs were differentially expressed in PC-3 spheres and adherent cells, we compared miRNA expression profiles using a miRNA microarray. We observed the increased expression of 25 miRNAs and decreased expression of 36 miRNAs in PC-3 sphere cells compared with adherent cells (Table 2). Third, to confirm our microarray data, qRT-PCR was performed to analyze the expression of the most significantly differentially expressed miRNAs (Table 2). The expression of miR-143 was down-regulated 8.4-fold in PC-3 sphere cells compared with adherent cells (Figure 1B). Next, we tested 10 miRNAs for which the expression levels were most changed during PC-3 sphere cells re-adherent culture on days 0, 2, and 4 by qRT-PCR. The expression of miR-143 was progressively increased during re-adherent culture, but no significant change was observed for the other 9 miRNAs (Figure 1C). Therefore, we selected miR-143 to further investigate its role in prostate cancer. These results suggested that miR-143 might play a regulatory role in prostate cancer stem cells differentiation in vitro.

**Table 2:** Differentially expressed miRNAs in PC-3 sphere cells of prostate cancer compared with PC-3 adherent cells by miRNA microarray and qRT-PCR

**Down-regulation**	**Fold change**		**Up-regulation**	**Fold change**	
Genes	Microarray	qRT-PCR	Genes	Microarray	qRT-PCR
hsa-miR-143	10.3	8.4 ± 0.57	hsa -miR-202	11.8	7.9 ± 1.84
hsa -miR-19a*	6.8	7.1 ± 1.13	hsa -miR-96*	8.7	5.2 ± 2.37
hsa -miR-125b	6.4	5.8 ± 0.79	hsa -miR-377	3.9	4.0 ± 0.39
hsa -miR-16	3.2	3.8 ± 1.61	hsa -miR-802	4.7	3.7 ± 0.84
hsa -miR-675	2.2	3.1 ± 0.42	hsa -miR-606	4.4	3.2 ± 0.68
hsa -miR-92a	4.3	2.9 ± 0.73	hsa-miR-142	3.1	3.1 ± 0.32
hsa -miR- let-7i	5.1	2.8 ± 0.32	hsa-miR-590	4.1	3.0 ± 0.53
hsa -miR-34a	3.0	2.7 ± 0.29	hsa-miR-376c	3.4	2.8 ± 0.32
hsa -miR-616	2.9	2.7 ± 0.65	hsa-miR-19a	3.0	2.7 ± 0.34
hsa -miR-933	2.9	2.5 ± 0.51	hsa-miR-381	2.4	2.7 ± 0.54
hsa -miR-17	3.7	2.4 ± 0.27	hsa-miR-19b	2.8	2.5 ± 0.42
hsa -miR-365	2.6	2.4 ± 0.39	hsa-miR-28	3.2	2.4 ± 0.57
hsa -miR-135b*	2.6	2.3 ± 0.41	hsa-miR-488*	3.0	2.4 ± 0.38
hsa -miR-939	3.4	2.3 ± 0.69	hsa-miR-18b	2.1	2.3 ± 0.42
hsa-miR- let-7c*	2.4	2.2 ± 0.28	hsa-miR-137	2.7	2.3 ± 0.63
hsa -miR-24	2.3	2.1 ± 0.33	hsa-miR-216b	2.6	2.2 ± 0.28
hsa -miR-10a	2.3	2.1 ± 0.45	hsa-miR-337	2.0	2.1 ± 0.23
hsa -miR-205	4.2	2.0 ± 0.25	hsa-miR-30a	2.3	2.0 ± 0.45
hsa -miR-584	2.8	2.0 ± 0.49	hsa-miR-452*	2.3	1.8 ± 0.25
hsa -miR-125a	2.5	1.9 ± 0.39	hsa-miR-570	2.2	1.4 ± 0.39
hsa -miR-574	2.3	1.8 ± 0.64	hsa-miR-301b	2.1	1.3 ± 0.52
hsa -miR-361	2.2	1.7 ± 0.44	hsa-miR-379	2.1	1.0 ± 0.35
hsa -miR- let-7 g	2.2	1.7 ± 0.65	hsa-miR-22	2.0	0.9 ± 0.56
hsa -miR-885	2.2	1.5 ± 0.34	hsa-miR-545*	2.0	0.7 ± 0.44
hsa -miR-325	2.1	1.4 ± 0.39	hsa-miR-922	2.0	0.5 ± 0.29
hsa -miR-9*	2.1	1.3 ± 0.52			
hsa -let-7c*	2.1	1.3 ± 0.26			
hsa -miR-27b	2.0	1.2 ± 0.44			
hsa -miR-296	2.3	1.1 ± 0.35			
hsa -miR-630	2.0	1.1 ± 0.22			
hsa-miR-138	2.0	1.0 ± 0.39			
hsa-miR-936	2.0	1.0 ± 0.35			
hsa-let-7e	2.0	1.0 ± 0.55			
hsa-miR-513	2.0	0.9 ± 0.51			
hsa-miR-135b*	2.1	0.9 ± 0.24			
has-miR-845	2.0	0.8 ± 0.19			

### PC-3 sphere cells migration was gradually enhanced in differentiation

**Figure 2:**
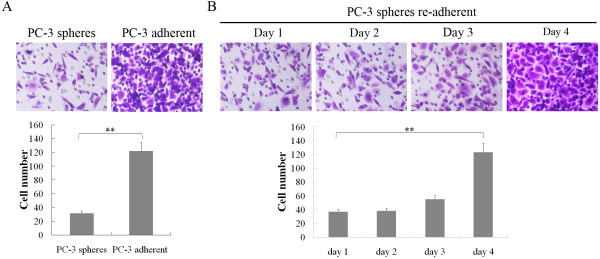
**The migration of PC-3 sphere cells was gradually enhanced in differentiation.A**, The migration capacity of PC-3 sphere cells and adherent cells was analyzed by transwell assays. Five predetermined fields were photographed at 200x magnification, and cells were counted and analyzed with a histogram (31.5 ± 3.5 versus 122.0 ± 12.5, Student’s *t* test). **B**, The migration capacity of re-cultured sphere cells on days 1, 2, 3, and 4 was tested by transwell assays. (ANOVA) **p < 0.01.

To evaluate the metastatic mechanism of prostate cancer stem cells, we compared the migration capacity of PC-3 spheres and adherent cells with a transwell assay. Interestingly, less PC-3 sphere cells penetrated through the gel-membrane compared with adherent cells (Figure 2A). However, when we digested the sphere cells into single cells for re-adherent culture, the cells gradually showed increased migration capability and reached the level of adherent cells on the fourth day (Figure 2B). These data suggested that prostate cancer stem cells might exhibit lower metastatic ability but generate differentiated cells expressing a highly aggressive phenotype.

### Down-regulation of miR-143 inhibited prostate cancer cells migration and invasion in vitro

**Figure 3:**
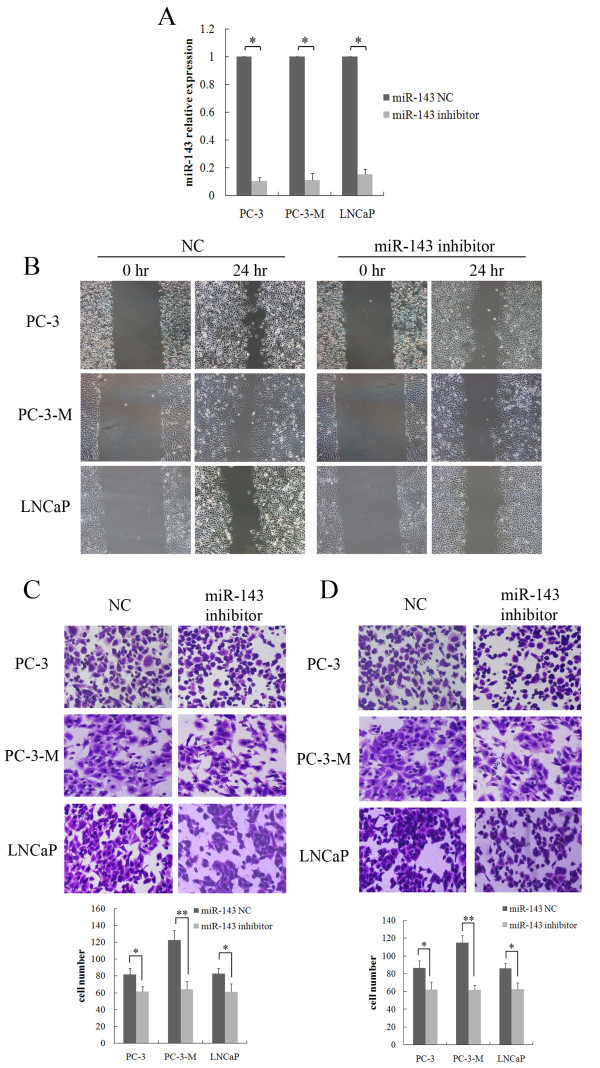
**The down-regulation of miR-143 inhibited prostate cancer cells migration and invasion in vitro.A**, The down-regulation of miR-143 in PC-3, PC-3-M and LNCaP cells by siRNA was verified by qRT-PCR. **B**-**C**, The migration of cells in which miR-143 was down-regulated was analyzed by a wound-healing assay and transwell assays. **D**, The invasive properties of the indicated cells were evaluated in an invasion assay using a transwell insert coated with Matrigel. Penetrated cells were counted and analyzed with a histogram (* P < 0.05, ** P < 0.01, Student’s *t* test).

MiR-143 expression and cells migration were increased during sphere cells differentiation. To evaluate whether miR-143 played an important role in the metastasis of prostate cancer cells, we decreased the expression of miR-143 in PC-3, PC-3-M and LNCaP cells by transient transfection with siRNA. MiRNA negative control-transfected cells, PC-3/NC, PC-3-M/NC and LNCaP/NC, were used as control groups. As shown in Figure 3A, the relative expression of miR-143 in PC-3, PC-3-M and LNCaP cells transfected with the miR-143 inhibitor was approximately 10-fold lower compared with cells transfected with NC. Migration and invasion assays were performed in vitro. Intriguingly, in wound-healing and transwell assays, cells migration was much slower and fewer cells penetrated through the gel-membrane when cells transfected with the miR-143-inhibitor compared with NC (Figure 3B,C). Moreover, cells invasion was markedly inhibited by the transfection with the miR-143 inhibitor (Figure 3D). These data strongly suggest that inhibition of miR-143 repressed the migration and invasion of prostate cancer cells in vitro.

### Down-regulation of miR-143 repressed PC-3-M metastasis in vivo

**Figure 4:**
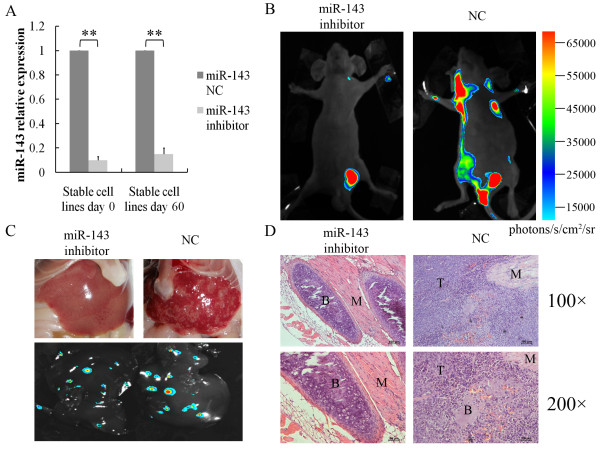
**The down-regulation of miR-143 repressed PC-3-M metastasis in vivo.A**, The down-regulation of miR-143 in PC-3-M stable cell lines on days 0 and 60 by shRNA was verified by qRT-PCR. **B**, Systemic metastases of PC-3-M cells was measured in mice by bioluminescence imaging 30 days post-implant. **C**, The liver metastasis of PC-3-M cells was measured by macroscopic and bioluminescent methods after autopsy. **D**, Histologic analysis of metastatic lesions in the ribs of nude mice was carried by H&E-staining in which tumors were marked as “T”, bone was “B” and muscle was “M”.

To further explore the effect of miR-143 on prostate cancer metastasis in vivo, PC-3-M cells were stably transfected with NC or miR-143 inhibitor and subcutaneously injected into nude mice. As shown in Figure 4A, the relative expression of miR-143 in PC-3-M cells transfected with the miR-143 inhibitor was stably about 10-fold lower compared with cells transfected with NC at least 60 days. The development and metastasis of tumors in vivo were monitored by visualizing the bioluminescence emitted from the luciferase-tagged tumors on day 30. Mice injected with the miR-143 inhibitor PC-3-M cells developed fewer systemic metastasis (2/10 versus 8/10), especially fewer macroscopic and bioluminescent nodes in the liver (3/10 versus 9/10), compared with mice implanted with NC cells (Figure 4B,C, Table 3). Histological confirmations were made by H&E-stainning (Figure 4D). Mice injected with the miR-143 NC PC-3-M cells developed severe metastatic lesions in the ribs of nude mice, and only a litter bone and muscle were residual. However, no bone metastasis was seen in the mice implanted with miR-143 inhibitor cells. These findings suggested that the down-regulation of miR-143 inhibited prostate cancer cells metastasis in vivo.

**Table 3:** Incidence of systemic and liver metastasis in transplanted nude mice treated with miR-143 inhibitor or NC

	**Systemic metastasis**	**Liver metastasis**
NC	8/10	9/10
MiR-143 inhibitor	2/10	3/10
***p *(***χ*^2^ test)	0.023*	0.02*

*, p < 0.05.

### MiR-143 targeted oncogene FNDC3B

**Figure 5:**
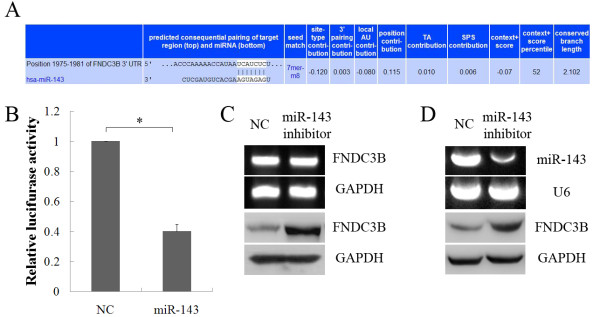
**MiR-143 targeted oncogene FNDC3B.A**, The putative miR-143-binding sequence was in the 3^′^-UTR of FNDC3B mRNA. **B**, The dual luciferase report assay of HEK-293 T cells cotransfected with psi-CHECK2-FNDC3B and miRNA (miR-143, NC). The Renilla luciferase signals were normalized to the internal firefly luciferase transfection control. **C**, The mRNA and protein expression of FNDC3B in PC-3-M cells transfected with miR-143 inhibitor or NC was analyzed by RT-PCR (above) and western blotting (below). **D**, The expression of miR-143 and FNDC3B in the tumor tissues of nude mice were analyzed by RT-PCR (above) and western blotting (below). U6 and GAPDH were internal control. (* P < 0.05).

To elucidate the mechanisms through which miR-143 induced prostate cancer cells metastasis, two publicly available algorithms were used to help predict miR-143 targets. Among the approximately 200 candidate genes, fibronectin type III domain containing 3B (FNDC3B) was a high-scoring candidate. FNDC3B is a member of the fibronectin family, which regulates cell motility, and is down-regulated in tumor cells with high metastatic potential [19]. Zhang et al. [20] reported that up-regulated miRNA-143 enhanced hepatocarcinoma metastasis by repressing FNDC3B expression. Thus, we focused on the possible regulation of FNDC3B by miR-143. As shown in Figure 5A, miR-143 was partially complementary to the FNDC3B mRNA 3^′^ untranslated region (UTR) element. Consequently, a luciferase reporter assay was performed to verify whether miR-143 could directly target the FNDC3B 3^′^-UTR. The luciferase activity of psi-CHECK2-FNDC3B was obviously decreased in the presence of miR-143, whereas no significant reduction was observed when cells were cotransfected with NC (Figure 5B). Moreover, we evaluated the effects of miR-143 on FNDC3B mRNA and protein levels in PC-3-M cells by RT-PCR and western blotting. FNDC3B mRNA levels showed no significant change whereas protein levels were increased in PC-3-M cells transfected with miR-143-inhibitor (Figure 5C). We also investigate the expression of miR-143 and FNDC3B in the tumor tissue of nude mice by RT-PCR and western blotting. As expected, the expression of miR-143 was significantly decreased whereas FNDC3B protein levels were enhanced in the tumor tissue of the group of miR-143-inhibitor (Figure 5D). Taken together, these findings suggested that miR-143 might modulate prostate cancer metastasis by targeting FNDC3B.

## Discussion

Cancer stem cells (CSCs) are involved in tumor progression and metastasis and are associated with increased aggressiveness and metastasis in vivo [5,12]. In our previous study, we enriched prostate cancer stem cells from PC-3 sphere cells in serum-free suspension culture and characterized their CSCs properties [7]. Several published reports have demonstrated that non-adherent spheres culture is increasingly used as an effective method to enrich and identify stem cells or putative CSCs [8,11,21]. Thus, we used spheres as a prostate cancer stem cells model to elucidate its metastatic mechanisms. Moreover, the expressions of cancer stem cells markers, such as Oct4, Sox2 and Nanog were gradually decreased when the sphere cells were digested into single cells for re-adherent culture. Interestingly, our results showed that sphere cells exhibited lower migration in a transwell assay, but the migration capability was gradually increased during the sphere cells differentiation. In fact, when growth factors were removed and the cells were exposed to 10% FBS-containing medium, sphere cells gradually became adherent, flat monolayer cells, which demonstrated the parental cell phenotype [11,22]. A recent study revealed that prostate cancer stem cells were able to generate differentiated cells expressing either a high- or low-level aggressive phenotype in vitro [22]. Taken together, our findings suggest that prostate cancer stem cells may exhibit lower metastasis but generate highly aggressive cells after differentiation in vitro.

In this study, we found that miR-143 was decreased in prostate cancer stem cells and progressively increased during the sphere cells differentiation. Furthermore, the down-regulation of miR-143 inhibited prostate cancer cells migration and invasion in vitro and metastasis in vivo. However, our results are inconsistent with some reports that miR-143 was down-regulated in several cancer tissues compared to normal tissue and was identified as a tumor suppressor [17,23,24]. A recent study showed that miR-143, miR-16 and miR203 modulated cell proliferation and manifested tumor suppressive effects in ER positive breast cancer [23]. The overexpression of miRNA-143 in bladder cancer cells significantly inhibited cell proliferation and RAS protein expression [24]. Bin X et al. [17] showed that miR-143 inhibited prostate cancer cells proliferation and migration through suppressing KRAS.

Conversely, some reports found that miR-143 was overexpressed in cancer samples [25,26]. A recent study revealed that miR-143 and miR-145 were overexpressed in invasive subpopulations of glioblastoma, and their down-regulation abrogated invasion [27]. Moreover, it was demonstrated that the up-regulation of miR-143 expression in hepatocellular carcinoma (HCC) promoted cancer cells metastasis by repressing FNDC3B. Overexpression of miR-143 promoted HCC local liver metastasis and distant lung metastasis in nude mouse model, but metastasis could be significantly inhibited by blocking miR-143 [20]. These results are consistent with our findings that miR-143 promotes prostate cancer cell metastasis by targeting FNDC3B. Taken together, this discrepancy could be due to the multifunctional nature of miRNAs. These findings indicate that miR-143 has the potential to regulate cell biology by modulating the expression of different target genes. Furthermore, the expression of miR-143 and FNDC3B in human clinical prostate cancer specimens is still unclear. Further investigation on the relationship between their expression level and metastasis, and its potential significance in prostate cancer metastasis, is underway in the laboratory.

## Conclusion

It is our novel discovery that miR-143 was up-regulated during the differentiation of prostate CSCs and promoted prostate cancer metastasis by repressing FNDC3B expression. Our results suggested that miR-143 might play a critical role in prostate cancer differentiation, which may generate highly aggressive cells to promote metastasis, and provide a potential development of a new approach for the treatment of metastatic prostate cancer.

## Abbreviations

CSCs: Cancer stem cells; FNDC3B: Fibronectin type III domain containing 3B.

## Competing interests

The author(s) declare that they have no competing interests.

## Authors’ contributions

XF and XC performed the initial experimental design, participated in the experiment, performed data analysis, and wrote the initial manuscript. GZ performed the animal experiments, and WD designed the methodology for miRNA target analysis. TL and QC designed and coordinated the study, analyzed data, and wrote the manuscript. All authors read and approved the final manuscript.

## Pre-publication history

The pre-publication history for this paper can be accessed here:

http://www.biomedcentral.com/1471-2407/13/61/prepub
